# Polymeric Materials for Hemostatic Wound Healing

**DOI:** 10.3390/pharmaceutics13122127

**Published:** 2021-12-09

**Authors:** Suvash Ghimire, Pritha Sarkar, Kasey Rigby, Aditya Maan, Santanu Mukherjee, Kaitlyn E. Crawford, Kausik Mukhopadhyay

**Affiliations:** 1 Department of Materials Science and Engineering, University of Central Florida, Orlando, FL 32816, USA; sghimire@knights.ucf.edu (S.G.); pritha@Knights.ucf.edu (P.S.); rigbyk@Knights.ucf.edu (K.R.); admaan@Knights.ucf.edu (A.M.); smukh1984@gmail.com (S.M.); 2 Department of Chemistry, University of Central Florida, Orlando, FL 32816, USA; 3 NanoScience Technology Center, University of Central Florida, Orlando, FL 32816, USA; 4 Biionix Cluster, University of Central Florida, Orlando, FL 32816, USA

**Keywords:** polymer, hemostasis, blood, wound, nanoparticles, metals, hydrogels

## Abstract

Hemorrhage is one of the greatest threats to life on the battlefield, accounting for 50% of total deaths. Nearly 86% of combat deaths occur within the first 30 min after wounding. While external wound injuries can be treated mostly using visual inspection, abdominal or internal hemorrhages are more challenging to treat with regular hemostatic dressings because of deep wounds and points of injury that cannot be located properly. The need to treat trauma wounds from limbs, abdomen, liver, stomach, colon, spleen, arterial, venous, and/or parenchymal hemorrhage accompanied by severe bleeding requires an immediate solution that the first responders can apply to reduce rapid exsanguinations from external wounds, including in military operations. This necessitates the development of a unique, easy-to-use, FDA-approved hemostatic treatment that can deliver the agent in less than 30 s and stop bleeding within the first 1 to 2 min at the point of injury without application of manual pressure on the wounded area.

## 1. Introduction

The hemostatic system of a multi-cellular organism is one of the most specialized and unique mechanisms to exist in nature, as it is the chief therapeutic mechanism initiated by the body to regulate blood loss in the occurrence of injury. This system is subsisted by all macroscopic, multicellular organisms, from the tiny zebrafish to humans [1]. Nonetheless, when the trauma/injury suffered is too severe, the body’s inherent hemostatic system falls short in stanching the profuse blood loss effectuated, which often leads to life-threatening situations [1,2]. This is especially true when wounds are on a massive scale and major arteries are affected [3]. Blood transfusions after a significant blood-loss injury are generally the most direct treatment; however, unpredictable complications during surgery or intractable environments at accident sites can often render blood transfusions extremely difficult, time-consuming, or impossible in unsterile conditions, thereby causing an unwanted delay in hemostatic application [1,4,5]. Employing appropriate wound-dressing mechanisms is pertinent to healing. It can serve to save lives, as wound sites serve as breeding grounds for secondary pathogens, which may cause the wounds to fester and lead to the fatality of sepsis [6,7]. The mechanism of intercepting blood flow, i.e., hemostasis, is also important outside of wounds and injuries, and therefore considerable research has been carried out to develop hemostatic and wound-healing materials, systems, and processes [8,9,10,11,12]. Moreover, studies have shown that hemostatic agents can benefit hospitals and the medical industry economically [13,14]. This perspective, therefore, underscores the necessity of developing novel, stable, and robust hemostatic agents.

Within the human body, natural blood coagulation and hemostatic processes involve a rather complex mechanism comprising initial vasoconstriction to limit blood loss at the injury site, consequent aggregation of platelets, followed by multiple physicochemical signals and secretions of proteins known as ‘coagulation factors’ that facilitate the formation of “fibrin” via multiple intermediate steps and employing a precursor called fibrinogen [1,15]. Hemostasis is essentially brought about by the activation of platelets. Damage in the endothelium leads to exposure of collagen, Willebrand factors (vWF), and tissue factors at the injury site. The exposed collagen provides a site for platelet adherence, accumulation, and activation at injury site, whereas the tissue factors are responsible for producing thrombin and other coagulation factors. Thrombin subsequently breaks down fibrinogen into fibrin, which then undergoes self-assembly to form a fibrin mesh. This fibrin mesh forged at the injury site promotes the aggregation of platelets, consequently stopping the outflow of blood [16]. Inspired by this exceptional natural mechanism of hemostatic action in the human body, there has been appreciable success in developing hemostatic agents that mimic parts of this process to engineer artificial mechanisms, which have shown favorable results in preventing blood loss [17].

Hemostasis is an individualized process since the mechanism varies significantly among subjects in terms of the rate of hemostasis and its longevity; thus, there is a requirement to design hemostatic materials based on individual criteria—there exists no ‘one size fits all’ solution [18,19]. Individual immune systems can reject these hemostatic materials or lead to thrombosis from excessive clotting, which can be fatal [20,21]. To avert the risk of venous and arterial thrombosis, recent developments of unique hemostatic materials that not only arrest bleeding but can also actively prevent excessive blood clots [22]. Such hemostatic materials have found immense recognition from medics and paramedics for their utility in administering first-aid [23]. A significant amount of research has gone into the design, fabrication, and development of suitable hemostatic materials, especially for arterial injuries responsible for profound blood loss [16,24,25].

While the primary objective of a hemostatic dressing is to prevent blood loss caused by varying levels of wound severity, an “ideal” hemostatic agent would also be biocompatible and mitigate undesirable side effects such as thrombosis, inflammation, and embolism [1]. There are many kinds of hemostatic dressings in use for trauma treatment and surgery, such as—tourniquets, bandages, foams, powders, and composites to name a few [18,26]. Such dressings are designed to be lightweight, easy to store, with swift, hassle-free administration mechanisms. They should also be conforming on and around the wound, implementable to seemingly impracticable locations, *viz*. non-compressible wounds, and be smoothly removable from the wound-cavities while remaining intact [1,2].

Common hemostatic materials can be classified as biologically derived or synthetically made [20]. Some examples of biologically derived materials include albumin, collagen, gelatin, chitosan, and keratin. They are readily available, easy to apply, have a long shelf life, and are generally inexpensive. However, some of these biologically derived hemostatic materials are known to engender transmission of diseases [27]. There have been multiple reports confirming disease transmission and toxicity associated with the usage of biologically derived hemostatic agents like collagen and albumin [28,29].

Therefore, the FDA recommends following the appropriate mitigation methods, such as proper material characterizations, biocompatibility evaluation, in vivo evaluation, and non-clinical performance testing, to ensure there is no potential risk of disease or injury associated with these materials [30]. In addition, biologically derived hemostatic agents generally lack mechanical strength and adhesiveness. This issue necessitates the development of effective hemostatic materials possessing satisfactory adhesiveness and good mechanical strength while alleviating the risks of other negative medical reactions when introduced at the bleeding site. Favorably, synthetic polymer-based hemostatic materials have found considerable success in exhibiting excellent blockage, mechanical strength, and tissue adhesion properties [31,32]. Common examples of synthetic polymer-based hemostatic materials include polyesters, polycyanoacrylates, polyalkyleneoxides, polyethylene glycol, and polyurethane, to name a few.

The purpose of this review is to provide a comprehensive guide to state-of-the-art polymer-based hemostatic dressings in trauma and wound care and some of the advancements made in the field over the last two decades.

## 2. Polymer-Based Hemostatic Materials

A “good” hemostatic material is expected to be high-strength and biodegradable with enhanced tissue compatibility, as well as improved wound dressing properties [16,33,34]. The rat and the porcine model have been adopted extensively for studying the efficacy of these wound-healing and hemostatic systems, due to their inherent similarity with the human body system [35,36,37,38,39]. Developing novel anti-microbial wound healing materials coupled with hemostatic materials and dressings has also garnered considerable attention in civilian and military trauma treatment [40].

Fibrin was the first hemostatic material introduced in modern surgery in 1909 by Bregel to prevent hematomas during surgery with the aim of averting other consequential infections [41]. During the 1980s and 1990s, the popularity of hemostats (a surgical tool employed to control bleeding) increased rapidly as surgeons attempted to control blood transfusion treatments due to the steep cost involved with the procedure and the underlying threat of disease transmission. Products were launched during this period by many of the leading medical device manufacturers, such as Johnson & Johnson, Pfizer, Baxter, Ethicon, Integra Life Sciences, which have now commercialized GelFoam (Pfizer, New York, NY, USA), FloSeal (Baxter, Deerfield, IL, USA), Instat (Johnson and Johnson, New Brunswick, NJ, USA), Surgicel (Ethicon, Somerville, NJ, USA), Helistat (Integra Life sciences, Plainsboro, NJ, USA) to name a few [3].

### 2.1. Natural Hemostatic Polymers

**Figure 1:**
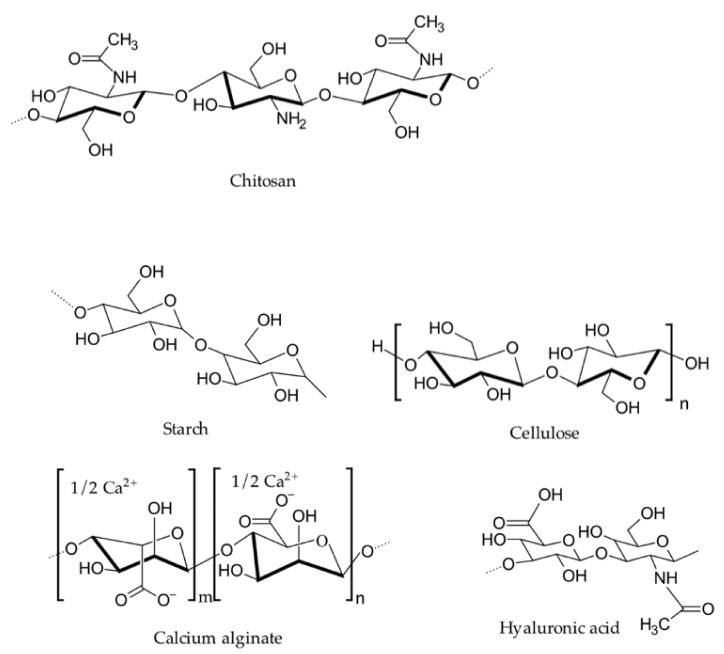
Representative chemical structures of naturally occurring hemostatic polymers.

One of the earliest hemostatic agents was the “antiseptic wax” formulated by Sir Victor Horsley in 1886 with salicylic acid, beeswax, and almond oil. Since then, researchers have made great strides in developing hemostatic agents, especially for use in combat casualty, surgical, and emergency settings. Figure 1 represents the chemical structures of some of the naturally occurring hemostatic polymers discussed in this section [42].

#### 2.1.1. Chitosan

Chitosan is a natural linear polysaccharide consisting of randomly distributed d-glucosamine and *N*-acetyl-d-glucosamine derived from chitin’s enzymatic or alkaline deacetylation [16]. Chitin is the second most abundant polysaccharide in nature and is found in the skeletons of insects, crustaceans, cell walls of fungi, and bacteria [43]. When the deacetylation of chitin is more than 50%, it is referred to as chitosan [44]. Chitosan is one of the most widely used natural polysaccharides in the food, agriculture, textile, polymer, and biomedical industries because of its attractive properties, such as biodegradability, non-toxicity, antibiosis, non-antigenicity, antimicrobial activity, and hemostatic ability [45,46]. The human use of chitosan dates back to the early 18th century; however, its application in the biomedical field was not realized until the mid-19th century. Subsequently, chitosan has been adopted in the fabrication of films, fibers, hydrogels, solutions, and composites for biomedical applications, including hemostasis [47].

Chitosan serves as an excipient in pharmaceutical formulations. It facilitates hemostasis through electrostatic interactions between its positively charged amino group and negatively charged membranes of red blood cells (RBCs), which form a strong hemostatic plug at the site of injury [46]. Chitosan also plays an instrumental role in platelet adhesion, activation, and aggregation by increasing the glycoprotein complex (GPIIb/GPIIa) and intracellular calcium ([Ca^2+^]_i_) mobilization [48].

The reactive amino group of chitosan can be favorably modified for different applications. Research has established that modified chitosan can enhance hemostatic efficacy [49]. Nie et al., have developed a chitosan-based hydrogel adhesive sealant consisting of thiolated chitosan (CSS) and maleimide-modified *ε*-polylysine via Michael addition crosslinking, which indicated adhesive strengths of up to four times that of their non-functionalized un-crosslinked counterparts [50]. The gelation time of the thiol-modified chitosan hydrogel prepared by fast in situ crosslinking by an efficient polypeptide crosslinker (CSS/EPLM) was found to be 15–215 s, depending on the chitosan concentration. Lab rodents were made use of as an experimental model to study the results. After the application of the hydrogel, blood loss from the injured liver in the rodent was seen to significantly drop from 106.7 to 28.3 mg. The decrease in blood loss was attributed to the appreciable hemostatic adhesion effect of the functionalized chitosan hydrogel [50]. Sundaraman et al., have developed a shear thinning chitosan-nano-bioglass (nBG) composite injectable hydrogel for effective bleeding control [51]. The incorporation of nano-bioglass (nBG) effectively boosted the hydrogel’s clotting capacity, as the silica, calcium, and phosphorous constituents of the nBG-activated coagulation factor XII (the coagulation factor secreted by the liver) as well as the intrinsic and extrinsic coagulation cascades. The in vivo hemostatic efficacy of 2% chitosan and 2% chitosan–5% nBG hydrogels were studied with respect to liver and femoral artery injuries in Sprague Dawley rat models. Both the in vivo and in vitro results of the prepared hydrogel suggested that the 2% chitosan–5% nBG had a more robust and faster blood-clotting mechanism as compared to the 2% chitosan hydrogel. The efficacy of the prepared hydrogels was tested in a femoral artery injury model in a rat. It was found that the stable blood clot formation time decreased from 387 ± 9 s to 318 s ± 7 s to 185 ± 9 s when the wound was untreated with hydrogel, treated with 2% chitosan hydrogel, and treated with 2% chitosan–5% nBG hydrogel, respectively. The authors concluded that the superior blood-clotting ability of the 2% chitosan–5% nBG hydrogel could be related to the synergetic effect of chitosan and nBG when in contact with blood, wherein the chitosan stimulates the blood coagulation cascade and stops the bleeding [51]. The porous structure and high surface area of chitosan make it well-suited for hemostatic wound-dressing materials [16]. Moreover, studies have shown chitosan to be responsible for higher plasma antioxidant activity and lower levels of oxidative stress in humans [52]. Chitosan-based hemostatic materials have been extensively used in treating heavy bleeding wounds, *viz.* severe femoral artery bleeding and severe visceral injury [53,54]. When these hemostatic agents come in contact with the blood, the chitosan granules or powders swell and initiate the coagulation cascade, which results in effective blood clotting [16].

#### 2.1.2. Collagen/Gelatin

Collagen is the most abundant protein in the extracellular matrix (ECM) and is found in most connective tissues in mammals. Collagen plays a pivotal role in maintaining hemostasis. In ECM-mediated hemostasis, collagen provides sites for platelet adhesion, activation, and accumulation in the wound sites and similarly initiates the coagulation cascades, which sustain the hemostasis process [55]. In 1969, Hait and his coworkers were the first to observe that bovine collagen (BC) can bind to wound sites and promote hemostasis in the event of mild to moderate hemorrhage [44]. Thenceforth, research has progressed much further, and various BC-based hemostatic materials have found their way into the pharmaceutical market in the form of powders, sponges, patches, fibers, and hydrogels. Some of the commercially available collagen-based topical hemostats today are Avitene^®®^, Instat^®®^, Tachosil^®®^, Hilitene^®®^, and Hemopatch^®®^. Although BC-based hemostatic materials provide effective hemostasis, they may have harmful repercussions, posing an allergic/immunogenic risk to the body [16]. Therefore, research proceeded to develop a collagen-derived non-immunogenic hemostatic material called gelatin.

Gelatin as a hemostatic material has gained a lot of attention because of its excellent characteristics, such as low antigenicity, low cost, biocompatibility, and biodegradability [56]. Gelatin is commercially available as hemostatic wound-dressing materials, such as FloSeal^®®^ (Baxter, Deerfield, IL, USA) and Gelfoam^®®^ (Pfizer, New York, NY, USA) [16]. A denatured form of collagen, gelatin, can be obtained by the hydrolysis of collagen under heat. It retains the hemostatic properties of collagen and can absorb up to 40 times its weight in blood. Gelatin yields hemostasis not only by activating and aggregating blood platelets but also by blocking the gash, as it has the capacity of swelling up to 200% in vivo when in contact with blood [46].

The hemostatic effectuality of gelatin is complemented when combined with other suitable hemostatic materials, such as alginate, collagen, graphene oxide, transglutaminase, and many more. Chen et al., have synthesized an alginate/gelatin sponge integrated with curcumin-loaded electrospun fibers (CFAGS) in an attempt to prevent post-operative bleeding and tumor recurrence [57]. The alginate/gelatin sponge was seen to control the release of curcumin, which accumulates at the surgical site and inhibits the local tumor recurrence. The sponge showed better results compared to the commercially available gelatin-based hemostasis sponges. Furthermore, it was devised to be non-toxic and did not trigger any harmful reactions in the body’s tissues and internal organs [57]. By the same token, Chu et al., have developed a microbial transglutaminase crosslinked thrombin-loaded gelatin hydrogel, which acted as a hemostatic sealant in liver trauma inflicted on rat models [58]. They prepared the hydrogel by successfully exploiting the excellent swelling ability of gelatin to absorb large quantities of blood at the wound site and administrated prompt hemostasis by inhibiting the blood-clotting time to 1 min [58]. Likewise, drawing on the good water dispersal ability, pH sensitivity, and large surface area of graphene oxide [59], Guajardo and his team have developed a GO-gelatin aerogel hemostat [56]. The GO-gelatin hydrogel lays out an immaculate structure for the coagulation system and serves as a platform for the enzymatic activity of coagulation factor IXa. However, the writers claim that the negatively charged GO-gelatin aerogels showed better clotting performance than positively charged GO-gelatin aerogels [56]. There has been extensive research to develop a superior gelatin-based hemostatic material free from toxicity and contamination [60,61].

#### 2.1.3. Alginate

Alginate is a negatively charged polysaccharide comprised of alternating groups of mannuronic and guluronic residues [62]. The alginate is generally extracted from naturally occurring brown algae by treatment with an alkali solution such as NaOH [63]. Alginates are considered one of the most promising polysaccharides in the field of drug delivery, tissue engineering, and hemostatic wound dressing because of their characteristic properties, such as biocompatibility, low toxicity, low cost, and convenient gelation with divalent cations (e.g., Ca^2+^, Ma^2+^, Ba^2+^, Sr^2+^) [64]. Since World War II, calcium alginate has been a notable candidate for hemostatic wound-dressing applications [62]. The hemostatic activity of calcium ions is realized based on an ion exchange mechanism. Calcium alginate comes in contact with blood, initiating the ion exchange process between the calcium ions present in the alginate and the sodium ions in the blood. The exchanged calcium ions then activate the platelets, thereby escalating the blood coagulation activity. The merits of working with alginate are not only limited to its hemostatic promise, as it has also been seen to possess remarkable antibacterial properties. Alginate is a remarkable asset for sterile wound treatment, as it absorbs the fluids secreted and curbs the unfavorable accumulation of liquid at the site of injury [49].

Alginates have been fabricated in the form of hydrogels, fibers, granules, and tissue adhesives for hemorrhage control. In vivo and in vitro studies of alginate-based hemostatic materials have demonstrated improved performance in low to moderate hemorrhage conditions. For example, an alginate-poly (hexamethylene) biguanide (PHMB)-silver nanoparticle (AgNP)-based hemostatic dressing has shown superior hemostatic as well as antibacterial activity. The excellent swelling property of the alginate complements both the antibacterial and hemostatic virtues of the prepared hemostatic gauze [65]. Hama et al., have prepared a hemostatic material by mixing alginate powder, porous hydroxyapatite, a random copolymer of ethylene oxide and propylene oxide, that decreases the blood stanching time significantly [66]. In addition to that, alginate-based hydrogels, fibers, and tissue adhesives have been reported as satisfactory hemostatic wound-dressing materials [67,68,69].

#### 2.1.4. Oxidized Cellulose

Cellulose, a homopolysaccharide generally derived from the cell walls of green plants, is an inorganic compound composed of *β*-linked D-glucose units [70]. Oxidized cellulose, a denatured polysaccharide obtained by chemical modification of cellulose, has been considered as one of the most promising hemostatic dressing materials due to its high water-absorption capacity, high mechanical strength, low hemolysis rate, and unique chemical and biological properties [70,71]. Both oxidized cellulose (OC) [46] and oxidized regenerated cellulose (OCR) [72] have been in use as hemostatic wound-dressing materials for decades. The mechanisms of hemostatic activity of OC and OCR involve blood adsorption, interaction with proteins and platelets, and intrinsic and extrinsic activation of blood coagulation pathways [73]. The most well-known ORC hemostat is Surgicel^®®^ (Johnson and Johnson, New Brunswick, NJ, USA). Amit et al., have carried out a prospective, randomized, and controlled study in order to gauge the effectiveness of ORC patch Sugicel^®®^ as a hemostatic agent in thyroid surgery [74]. The findings indicate that the routine use of ORC hemostatic agents could have potentially harmful effects in thyroid surgery [74]. Another study indicates that a biodegradable hemostat synthesized by blending oxidized regenerated cellulose and chitin/chitosan can impart decent antimicrobial as well as hemostatic properties in the treatment of chronic infections in farm animals [75]. Similarly, an ORC-modified chitosan foam sponge also exhibited rapid hemostasis on the mouse tail vein and rabbit femoral artery model (in vivo and in vitro) [70]. However, despite the rapid-response results observed on testing oxidized regenerated cellulose [72], Lewis et al., have carried out a comparative study between ORC and ONRC (oxidized non-regenerated cellulose) as hemostatic agents and concluded that the ONRC presents superior hemostasis and equivalent bactericidal effectiveness as compared to the ORC [76]. The comparative study was carried out using Traumastem^®®^ (ONRC, Biostar, New Taipei, Taiwan) and Surgicel^®®^ (ORC, Johnson and Johnson, New Brunswick, NJ, USA). The in vivo study was characterized and the rates of achieving hemostasis and hemostatic success were noted by implementing a non-heparinized porcine liver abrasion model and a peripheral vascular surgery heparinized leporine femoral vessel bleeding model [76].

#### 2.1.5. Dextran

Dextran is a complex polysaccharide, specifically a branched poly-α-d-glucoside of microbial origin endowed with high water-absorption properties, making it suitable for hemostatic functions. Inorganic agents can oxidize the diols present in dextran to aldehydes, which readily crosslink with tissues and proteins, inducing adhesion and coagulation. In an experimental study, Sigurjonsson et al., have found that albumin generally administered to patients undergoing gynecological surgery can effectively be substituted by dextran to induce greater plasma volume expansion, and hence better coagulation [77]. Dextran can also be functionalized to act as sealants in the form of sponges or hydrogels. Liu et al., have developed an in situ aldehyde dextran (PDA) sponge possessing strong tissue adhesion, low cytotoxicity, and excellent biodegradability [78]. The sponge was tested on rabbit and pig models and the clotting kinetics suggested that it could absorb up to 20 times more fluid compared to other generic hemostats such as chitosan. Some unconventional attempts of functionalizing dextran for hemostasis applications have also been carried out. For example, Wang et al., have synthesized a form of tissue adhesive with oxidized urethane dextran (Dex-U-AD) and gelatin [79]. A photopolymerization process was implemented to induce double crosslinking. From their findings, they reported high adhesion strength and also suggested its potential application as a novel tissue.

#### 2.1.6. Hyaluronic Acid

Hyaluronic acid, a high molecular weight polymer of extracellular matrix (ECM) comprising alternating units of D- glucuronic acid and N-acetylglucosamine, is yet another polymer that has found its place in the list of noteworthy constituents in hemostatic materials. [46,80,81]. HA increases cell adhesion and cell migration by virtue of its excellent water retention and inherent swelling properties, which sustain favorable conditions for hemostasis and wound healing [82]. In vivo and in vitro studies of hyaluronic acid as a hemostatic hydrogel have found remarkable results, unlocking its potential and practicality in wound dressing materials.

**Figure 2:**
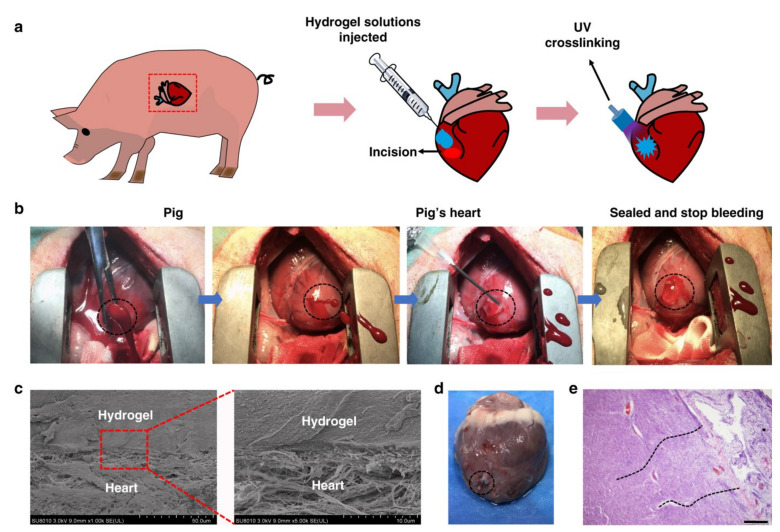
Selective results for hemostatic properties of GelMA:HA-NB matrix hydrogel prepared using rapid polymerization using UV light radiation. (**a**) Schematic showing a surgical procedure in a pig cardiac puncture injury model. (**b**) Images showing the real surgical procedure, application of hydrogel, and hemostatic efficacy of hydrogel, where hydrogel stops bleeding completely within 30 s in a 6 mm pig cardiac injury. (**c**) SEM images showing the interface between hydrogel and pig’s heart obtained from immediate postoperative autopsy on the heart of a pig. (**d**,**e**) Heart autopsy and tissue staining images of the interface between hydrogel and hearts of a pig after two weeks of post-operative recovery. Adapted with permission from [32]. Copyright © 2019, Springer Nature.

Hung et al., have developed a photoreactive adhesive hydrogel composed of GelMA (methacrylated gelatin)-HA-NB (glucosaminoglycans hyaluronic acid), which undergoes rapid gelling under UV light radiation and effectively seals the bleeding arteries and cardiac walls [32]. The prepared hydrogel (GelMA/HA-NB with LAP polymerization initiator) was able to withstand pressures of up to 155 ± 27 mmHg, which is higher than the average normal systolic blood pressure [32]. Based on their results, the authors suggested that the adhesive hemostatic hydrogel can be used to treat high-pressure bleeding, such as arterial and cardiac hemorrhages [32]. Figure 2 shows the hemostatic results obtained from the application of the GelMA:HA-NB gel matrix in a cardiac penetration injury inflicted on a pig. Figure 2a,b illustrate the schematic and surgical procedure of the cardiac puncture, hydrogel application, and UV light irradiated to seal the wounded heart muscle. After UV polymerization, the bleeding subsided rapidly and completely ceased within 30 s for a 6 mm internal diameter cardiac wound. Figure 2c represents the scanning electron microscopy image, showing the adhesion of the matrix gel with the cardiac tissue, which was obtained after the immediate postoperative autopsy of the pig’s heart [83]. The heart autopsy and tissue staining images obtained 2 weeks after the surgery indicate the effectual sealing of the wound [32]. In addition to its hemostatic properties, the hyaluronic acid-based hydrogel has also exhibited considerable promise in other functionalities, such as antibacterial, adhesiveness, and radical scavenging activities. Liang et al., have developed an antioxidant-conductive, photothermal, antibacterial, hemostatic hydrogel composed of hyaluronic acid-graft-dopamine and reduced graphene oxide (rGO) using the H_2_O_2_/HPR (Horseradish peroxidase) system. The prepared hydrogel improves the tissue thickness and collagen deposition, thereby providing rapid hemostasis treatment, which was shown to be more efficient than the commercial Tegaderm films [84]. In vitro and in vivo studies of hemostats prepared by polymer peptide interfusion (HAPPI) were investigated by Gao et al. [59]. The in vitro studies showed that the HAPPI selectively binds to the activated platelets, which increases the accumulation of activated platelets to the wound sites. Similarly, in vivo studies of HAPPI exhibited decreased blood loss and reduced bleeding time in the mouse vein laceration model [59]. Hence, the authors suggested that the HAPPI hydrogel could be applied as a translatable intravenous hemostat [59]. Furthermore, hyaluronic acid can be productively modified with other hemostatic polymers such as chitosan and gelatin, as well as certain inorganic polyphosphates to form an effective tissue-adhesive hemostatic hydrogel [85,86]. Some of these hyaluronic acid-based hydrogels have also been shown to hold exciting prospects in drug release and tissue engineering applications, along with hemostasis [85,87,88].

#### 2.1.7. Starch

Starch is the most abundant natural polysaccharide in nature and can be derived from plants as a branched glucose polymer [89]. Starch has been widely used for biomedical applications such as tissue engineering and drug delivery for many years. Its availability, low cost, excellent biocompatibility and degradability, and high water-absorption properties make it an appealing constituent for engineering hemostatic wound-dressing materials [90]. There are many starch-based hemostatic agents which are capable of administering rapid hemostasis to uncontrolled and non-compressible severe hemorrhages. The high specific surface area and porous structure of starch help in inducing hemostasis by absorbing blood, aiding the accumulation of platelets, red blood cells, and coagulating factors at the injury site, which in turn accelerates the blood coagulation cascade [90]. In 2019, Panwar et al., developed a calcium-modified carboxymethyl starch (CaCM-starch) as a topical hemostat [91]. They studied the in vivo hemostatic efficacy of the CaCM-starch hemostat in a rat abdominal aorta and rat liver injury model. The carboxymethyl modification enhances the fluid absorption and swelling properties of starch, thereby boosting the overall efficacy of the system [91]. Wang et al., have played on the hemocompatibility and cytocompatibility of starch and went on to develop a sodium trimethaphosphate crosslinked starch/hyaluronic acid (ScSH) hemostat composite [92]. The hemostatic efficacy of ScSH was tested with both in vivo and in vitro experiments, and they were able to conclude that the addition of hyaluronic acid greatly influenced and increased the blood-clotting ability of the developed hemostat. Moreover, the hemostatic efficacy of ScSH composites was found to be similar to the commercially available hemostat Quickclean^®®^ [92]. Although starch has excellent hemostatic properties, it falls short on tissue adherence, rendering it unfit for specific clinical applications. In an attempt to alleviate this problem, inspired by muscle-adhesive protein, Cui et al., have synthesized a tissue-adhesive hydrogel composed of starch, succinic anhydride, and dopamine (St-Dopa hydrogel) [90]. Both in vivo and in vitro results of St-Dopa hydrogel showed better capacity as compared to chitin hydrogels. The rapid sol-gel transition, good swelling ratio, biodegradability, and excellent cyto/hemocompatibility make this hydrogel a promising candidate for tissue adhesion and as a hemostatic material [90]. It is important to note that mechanical strength and biodegradability are as crucial as the blood-clotting potency in designing hemostats. Tavakoli et al., have demonstrated that the kappa carrageenan coated on the starch/cellulose nanofiber hydrogel greatly increases the mechanical strength of the hydrogel without compromising on the clotting ability of the starch matrix [93]. Similarly, to address the increased clinical demands of hemostatic sponges for uncontrolled and non-compressible hemorrhage, Yang and his group have developed a peptide-immobilized starch/PEG sponge with rapid shape recovery [94]. They chemically immobilized thrombin-receptor-agonist-peptide (TRAP) on the starch/PEG sponge (TRAP-Sp). The prepared TRAP-Sp hemostat was tested by both in vivo and in vitro experiments. The in vivo experiment was carried out with the help of a rat femoral artery hemorrhage model for hemostatic efficacy, and a penetrating liver wound model for non-compressible hemorrhage testing. Both the in vivo and in vitro studies exhibited improved results for both uncontrolled and non-compressible hemorrhage [94].

Table 1 summarizes the mechanisms of action and findings on the natural polymeric systems discussed in this section.

### 2.2. Synthetic Hemostatic Polymers

#### 2.2.1. Polyester

Aliphatic polyesters such as polycaprolactone (PCL) and polylactic-co-glycolic acid (PLGA) have been extensively used in the biomedical field as tissue adhesives. These adhesives hold the tissues, act as sealants, and provide hemostasis [105]. One of the examples of PLGA based adhesive is TissuePatchDural^TM^ (TPD). TPD, a synthetic film made up of a laminated structure of glycolic acid, acts as a tissue sealant against fluids including cerebrospinal fluid (CSF) and blood [106]. TPD plays a key role in preventing postoperative complications associated with CSF leakage. Van der Brelie et al., have clinically examined the efficacy of TPD in preventing CSF leakage in 25 patients who underwent intradural neurosurgical procedures. The PLGA and tissue reactive polymer were seen to provide fast and strong chemical bonding to the patch, and it was concluded that the TPD is a safe, biodegradable, effective sealant for CSF leakage [107]. Similarly, exploiting its excellent sealing properties and biodegradability, Yuan et al., have developed hemostatic gauze scaffolds containing G-CSF (granulocyte colony-stimulating factor) loaded and PLGA coated dextran nanoparticles known to be beneficial for cancer chemotherapy, radiotherapy, and tumor resection [108]. The as-prepared hemostatic gauze scaffold improved the neutrophilic granulocyte level in blood during chemotherapy and displayed excellent hemostatic efficacy in Sprague Dawley rats (in vivo study) [108]. Fornaguera et al., have carried out an experiment to study the interaction of PLGA nanoparticles with two model blood proteins (Bovine serum albumin (BSA) and fibrinogen) [109]. It was concluded that loperamide loaded PLGA nanoparticles do not need any modification for intravenous administration whereas carbosilane cationic dendrons of second- generation (G2SN)- functionalized PLGA nanoparticles must undergo surface modification to be administered intravenously [109].

Similar to PLGA, PCL has also been applied as a tissue sealant for hemostatic applications. For instance, Jaiswal et al., have synthesized an electrospun gelatin-PCL nanofibrous matrix and tested its hemostatic efficacy using a rat liver model [110]. The in vivo and in vitro studies indicated that rapid hemostasis could be achieved while also enhancing its liver regeneration ability [110]. The composite scaffold made by sandwiching PCL in between layers of chitosan and gelatin (i.e., chitosan/PCL/gelatin) also displayed accelerated blood clotting and effective hemostasis properties [111]. As a result, it was concluded that the electrospun chitosan/PCL/gelatin composite scaffolds have the potential of imparting tissue regeneration properties in addition to hemostasis [111]. Furthermore, Giri dev and Hemamalini have been successful in developing a PCL/starch mat that carries up to 240% of the swelling ability as compared to a pristine PCL mat [112]. The PCL/starch mat showed impressive hemostatic potential with a higher blood clotting rate. The time to achieve hemostasis with the developed mat was found to be 156 s [112]. Similarly, in vivo and in vitro examination of PCL/CaCO_3_ nanofibers coated with *β*-chitosan showed better blood coagulation ability compared to PCL nanofibers [113]. These extensive studies performed have established that the polyesters are excellent biodegradable tissue adhesives having high hemostatic potential when engineered with other natural and synthetic hemostatic materials.

#### 2.2.2. Polyethylene Glycol (PEG)

PEG is a petroleum-derived polyether compound widely used in pharmaceuticals and medicine. Zhu et al., have studied a hyaluronic acid and PEG hybrid material for wound-healing purposes. The encapsulated “nanogel” they developed was then also loaded with chlorhexidine diacetate (CHX), and the authors have demonstrated superior wound-healing properties in-vivo through mouse models [114]. Orgill et al., have synthesized a PEG/microfibrillar collagen composite, which demonstrated good hemostatic performance while assisting in bone repair. The PEG and microfibrillar components exhibited better bone healing than in control samples with no composite added [115]. In another study, A PEG-coated collagen pad was developed for hemostatic applications by Lewis et al., The authors demonstrated total hemostatic performance, i.e., complete stoppage of bleeding within 10 min after application, indicating its superior performance [116]. The chitosan-poly (ethylene glycol)-tyramine (CPT) hydrogel prepared using the enzymatic crosslinking method has shown significant promise in hemostasis and wound healing. The CPT hydrogel was prepared by grafting tyramine-modified PEG onto the chitosan backbone [117]. The enzymatic crosslinking method increased the water solubility of the chitosan, which facilitated the synthesis of the hydrogel within 5 s of administration into the body. The prepared hydrogel showed up to a 3–20-fold increase in tissue adhesiveness compared to fibrin glue. In addition, the CPT hydrogel ultimately resolved the 1.5 cm long skin incision wound and skin thickness depth in rats within two weeks [117].

#### 2.2.3. Polycyanoacrylate

Polycyanoacrylate, best known as a key component in structural adhesives, also finds extensive functionality in medicinal uses. Its unique ability to undergo rapid polymerization in ambient conditions makes it an apt choice for hemostasis applications. Perez et al., have studied the role of *N*-butyl-2-cyanoacrylate as a tissue adhesive and hemostatic agent after oral surgery. The results indicated that considerable pain relief could be realized when these agents were applied in the areas of surgery, coupled with faster wound healing [118]. Biocompatibility and wound healing of a polymer-modified allyl-2-cyanoacrylate have been studied by Lee and his coworkers. Upon application at the wound site, the material was able to demonstrate significant wound-healing properties and reduced inflammation in the incised dermal tissue of rats, thereby proving their efficacy [119]. Poly cyanoacrylates have also been considered for intraoral wound closures in the form of isoamyl-2-cyanoacrylate. In an adhesive form equivalent to sutures, it demonstrates reduced swelling, painless application, and analgesic properties, thereby showing significant promise [102]. The strong interlocking bonds caused by the rapid curing of the polymer allow for significant strength enhancement as well. However, it should be noted that hydrolysis can expedite the degradation of the polycyanoacrylate chains, which generates toxic products such as formaldehyde and cyanoacetate. This causes inflammatory responses, which may result in wound infection. In addition, the degradation of the polymer renders its application for bandages designed for long-term applications [72,120].

**Figure 3:**
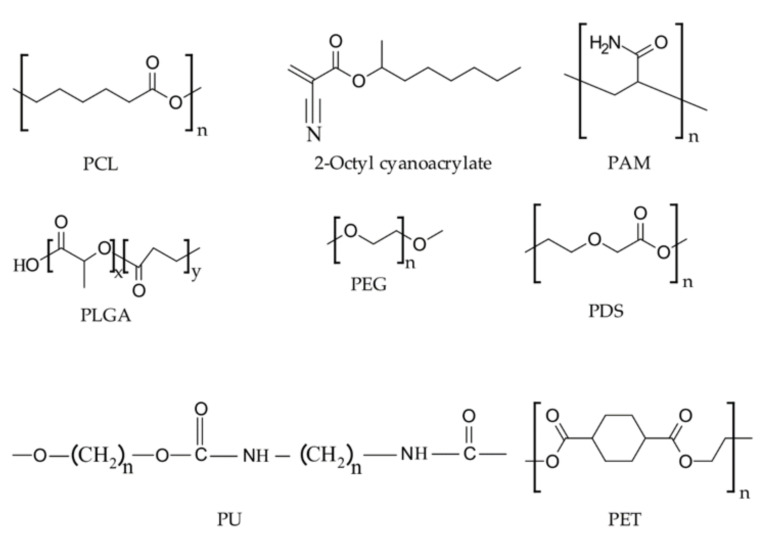
Representative chemical structures of synthetic hemostatic polymers.

Figure 3 illustrates the basic structures of some of the synthetic polymers discussed in this section.

#### 2.2.4. Polyurethane (PU)

Polyurethane is a synthetic polymer having a wide variety of applications in different fields [106]. In the biomedical field, PU has been used as hemostatic materials, vascular tissue graft sealants, bone fixation materials, scaffolds, etc. [121,122,123]. For its notable property of remaining functional in the absence of hemolysis, synthetic polyurethanes have been employed as a hemostatic material for a long time in the form of foams, films, and hydrocolloids. Polyurethane foam-based hemostatic materials are commercially available and have been used extensively in the medical industry. Some of the commercially available hemostatic materials are Tegaderm^TM^ (3M Science), TissuGlu^®®^ (Cohera Medical, Inc.), ResQFoam^TM^ (Arsenal Medical, Inc.), and Nanosan^®®^-Sorb (SNS Nano Fiber Technology, LLC.). Tegaderm^TM^, a semi-permeable membrane with an acrylic adhesive, is one of the most significant polyurethane-based hemostatic materials engineered [124]. Tegaderm^TM^ can be applied for hemostasis, wound healing, as well as for anti-bacterial applications. As a result of being optically transparent, Tegaderm^TM^ also provides visibility to the I.V. catheter insertion site. TissuGlu^®®^ is another PU-based hemostatic dressing tissue adhesive designed for acute to chronic wounds. It is the first FDA-approved tissue adhesive for internal use. Ohlinger et al., have studied the use of TissuGlu^®®^ for a mastectomy procedure with or without lymphonodectomy [125]. They concluded that the polyurethane-based tissue adhesive (TissuGlu^®®^) might serve as a less invasive alternative to the internal JP drain generally used for post-operative care after a mastectomy [125]. Similarly, Burnett et al., have carried out an experiment in a swine femoral artery injury model (in vivo) to study the hemostatic efficacy of a polyurethane-based hemostat (i.e., Nanosan^®®^-Sorb) and compared it with the commercial chitosan dressings (second generation HemCon^®®^) and cotton gauze [126]. Nanosan^®®^-Sorb hemostat was proven superior with an increased survival rate, increased mean arterial pressure, and decreased shock index compared to both the chitosan dressings [126].

Polyurethane has also been used to develop shape memory polymer (SMP) foam composite devices, which have demonstrated enhanced fluid uptake, bactericidal properties, and increased hemostatic ability [127]. The polyethylene glycol diacrylate–polyvinylpyrrolidone (PEGDA-PVP) hydrogel-coated SMP foam increased fluid uptake by 19 times as compared to an uncoated SMP foam. The bactericidal properties stem from the elemental iodine added to the hydrogel. Furthermore, the SMP foam composite device showed a 15-fold volume expansion when submerged in water at 37 °C for 15 min, while retaining the shape memory properties of the foam [127]. It showed no tissue damage when the expanded device diameter was larger than that of the targeted wound site [127]. The enhanced fluid uptake along with the bactericidal properties of the prepared SMP foam composite indicates its potential applicability in non-compressible hemorrhage as a hemostatic agent. ResQFoam^TM^ is an example of an injectable self-expandable polymer foam designed for the treatment of non-compressible hemorrhage. In 2010, Arsenal Medical in collaboration with Massachusetts General Hospital and Harvard Medical school developed a self-expandable polymer foam with an impressive capacity to treat major abdominal bleeding due to trauma [128]. This hemostatic polymer foam was specially designed for use at the point of care, especially for warfighters, and is regarded as a “bridge to surgery” [128]. These findings open up a new avenue for the usage of self-expandable polyurethane polymers for hemostatic applications, especially in non-compressible hemorrhage. Duggan et al., have carried out a study on the application of a self-expanding polyurethane polymer and found that the polyurethane polymer improves survival rate in non-compressible massive abdominal hemorrhage [129]. A lethal, non-coagulopathic, closed cavity, non-compressible, hepatoportal injury model was used in a 40 to 45 kg *Sus scrofa domestica* to investigate the hemostatic efficacy of a self-expandable polyurethane polymer. The conformal contact of the polymer to the injury site was seen to play a significant role in improving the survival rate of swine with hepatoportal injury [129]. Although most topical hemostatic agents expedite blood clotting, some of them may induce undesired inflammation and foreign body reaction, as noted earlier for polycyanoacrylates. This can lead to severe consequences in some cases, especially for patients with neurosurgical trauma as inflammatory responses often trigger secondary brain injury. To overcome this issue, Huang et al., have developed a hemostatic agent possessing anti-inflammatory properties. The hemostatic agent was prepared by incorporating carboxyl-functionalized biodegradable polyurethane nanoparticles (PU NPs) in a commercially available hemostatic gelatin powder (Spongostan^TM^) [85]. The prepared hemostat was applied on an injured male Sprague Dawley rat’s cortex. It was concluded that the PU NPs hemostatic agents have neuroprotective potential as the PU NPs-gelatin indicated suppressed pro-inflammatory M1 biomarkers, elevated anti-inflammatory M2 biomarkers, and reduced inflammatory cells at the sites of implementation, as compared to commercially available gelatin hemostats in a rat cortex model (in vivo) [85].

#### 2.2.5. PolySTAT

**Figure 4:**
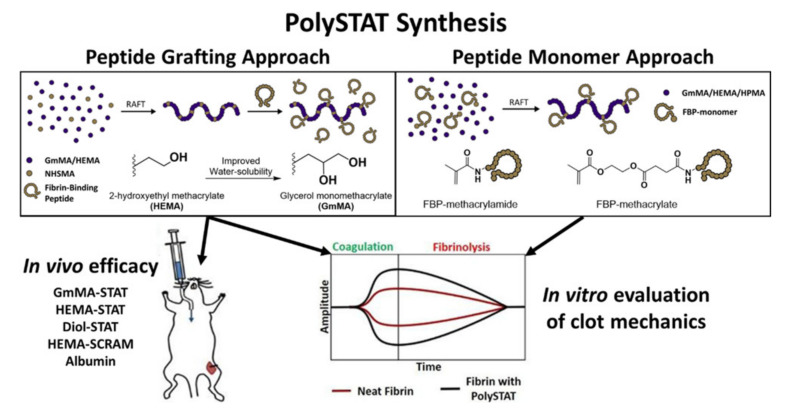
PolySTAT synthesis mechanism and its hemostatic performance. Adapted with permission from [134]. Copyright © 2020, American chemical society.

PolySTAT, a newly developed hemostatic polymer that mimics the properties of transglutaminase factor FXIII, is composed of a linear hydroxyethyl methacrylate grafted with fibrin binding peptides [130]. In 2015, inspired by the mechanism of transglutaminase factor FXIII, Chang et al., first developed a synthetic polymer, i.e., PolySTAT, which circulates innocuously in the blood, identifies the injury sites, and brings about hemostasis through fibrin crosslinking [131]. The authors carried out in vitro and in vivo experiments to determine the hemostatic efficacy of PolySTAT. The in vitro study under coagulopathic and hyper-fibrinolytic conditions showed that the PolySTAT accelerated the clotting kinetics, increased the clot strength, and decreased the clot breakdown rate [131]. Correspondingly, the in vivo study showed increased survival rate, decreased blood loss, and improved blood profusion in a mice femoral artery injury and blood resuscitation model [131]. Motivated by their findings, the authors proposed that the impressive properties and performance of PolySTAT (both in vivo and in vitro) make it an excellent hemostatic material for clinical translation [131]. Chang et al., further investigated the role of PolySTAT on the mechanical strength of clots during clot formation and breakdown using rotational thromboelastometry (ROTEM) [132]. The results suggested that the PolySTAT and fibrinogen both contribute significantly to promoting the formation of clots and improving clot stability. Against rFVIIa, PolySTAT showed similar blood coagulation rates but significantly higher clot firmness [132]. The blood coagulation kinetics, clot firmness, and clot breakdown efficacy were found to depend on the PolySTAT valency. The PolySTATs having a valency of four and higher showed increased clot firmness and formation rates with lower clot breakdown. Lamm et al., have shown that the quadrivalent PolySTATs increased clot firmness values by 57% and decreased the breakdown values by 69% as compared to phosphate-buffered saline [133]. Furthermore, Lamm et al., also carried out an experiment with PolySTAT to optimize its yield and synthesis method, which could potentially capacitate mass production of PolySTAT and its clinical translation [134]. The primary purpose of the research was to increase the water solubility and yield without compromising the structure and hemostatic efficacy of PolySTAT. From their study, they found that the substitution of hydroxyethyl methacrylate with glycerol monomethacrylate (GmMA) greatly increases the water solubility of PolySTAT without affecting its performance [134]. The hemostatic efficacy of the GmMA-based PolySTAT was verified by an in vivo study of a rat femoral artery bleeding model. Moreover, the ROTEM experiment suggested that the methacrylamide and methacrylate peptide monomer synthesized and polymerized via reversible additional fragmentation chain transfer greatly improved the PolySTAT yield [134]. Figure 4 represents the schematic of the PolySTAT synthesis mechanism and it’s in vivo and in vitro hemostatic efficacy [134]. The PolySTAT can also be used to increase the hemostatic efficacy of chitosan. Chang et al., have designed a PolySTAT/chitosan gauze and compared its efficacy to the commercially available chitosan gauze Celox^®®^ Rapid [135]. Although there is no significant difference in fiber size, morphology, and pore size of nonwoven PolySTAT/chitosan and Celox^®®^ Rapid, the PolySTAT/chitosan gauze demonstrated higher blood adsorption rates compared to Celox^®®^ Rapid in the rat femoral artery injury model [135]. Henceforth, PolySTAT has huge potential application as a hemostatic material for non-compressible hemorrhage.

#### 2.2.6. Other Synthetic Hemostatic Polymers

In addition to the commonly used synthetic hemostatic polymers such as PEG, PU, poly cyanoacrylate, and polyesters, other synthetic hemostatic polymers such as siloxane, polyethylene oxide (PEO), polyacrylamide (PAM), polyethylene terephthalate, and polydioxanone (PDS) have also been employed for hemostatic applications. These polymers have demonstrated excellent hemostatic performance alone or in combination with other natural or synthetic polymers. Mukhopadhyay et al., have developed a siloxane-based semi-solid matrix that acts as an artificial blockage to the bleeding site and can be applied to moderate to severe bleeding [136]. They were able to devise a formulation combining the two siloxane-based mixtures, in which the first component consists of a polymeric matrix, a surfactant, one or more filler compounds, and a metal compound, whereas the second component comprised a combination of polymer, a filler, a surfactant, and hydrogen peroxide [136]. By implementing the prepared hemostatic matrix, they were able to achieve a nearly 96% survival rate in a pig model. The authors suggest that the proposed siloxane-based hemostatic matrix is capable of arresting/restricting bleeding from internal and external wounds in humans and other animals by forming an artificial blockage to the wound site [136]. Similarly, Barba et al., have prepared an effective hemostatic granule dressing made of carboxymethyl cellulose, kappa carrageenan (KC), polyethylene oxide (PEO), and polyethylene glycol (PEG) by gamma radiation [137]. In the prepared granule composite, PEO helps in forming a mechanically stable interpenetrating network when mixed with KC. An in vitro study of the prepared hemostatic dressing indicated a low blood-clotting index, high platelet adhesion capacity, and accelerated clotting time [137]. Furthermore, using a femoral artery bleeding model in rats, the study also showed that hemostasis was achieved within 90 s with a 100% survival rate [137]. Drawing on the benefit of the good expandable property of polyacrylamide (PAM), Wang and his group have developed an expandable keratin hemostatic sponge by radical polymerization of keratin and PAM [138]. This sponge showed excellent hemostatic ability in both the rat penetrating liver hemorrhage model and the swine femoral artery transection hemorrhage model. Furthermore, the prepared expandable keratin sponge was successful in maintaining the mechanical strength of the liver tissue for up to 1 h after its application [138]. Li et al., have synthesized a superhydrophobic carbon nanofiber mat on a PDMS substrate, that not only limits blood wetting by virtue of its super-hydrophobicity but also promotes fibrin growth on the carbon fiber surface [139]. The enhanced surface roughness of the hemostat facilitates the presence of small air pockets at the region of contact with skin, allowing unforced clot detachment with minimal pain—a very sought-after feature in engineering novel hemostats [139]. Table 2 summarizes the various synthetic polymers discussed and the relevant findings from the research conducted to date.

Another synthetic polymer, polyethylene terephthalate, has been seen to show a hemostatic response when coated with heparin. Kolar and Primc have carried out an experiment to study the hemostatic response of PET treated with oxygen and nitrogen plasma afterglows [143]. The hemostatic response of PET was determined by analyzing the density of adhered blood platelets as well as their morphological properties [143]. The hemostatic response of the PET treated with oxygen and nitrogen plasma was found to be better than that of the untreated PET. In addition, the oxygen-treated polymer showed improved hemocompatibility compared to the nitrogen-treated polymer. The authors have hypothesized that the better performance of the oxygen-treated polymer might be because of the deactivation of the surface functional group upon incubation with heparin [143]. The development of improved hemostatic dressings is always in great demand. To address the issue related to hemorrhage during surgery, Boerman et al., have designed a hemostatic material based on NHS (N-hydroxysuccinimide ester)-functionalized poly 2 (oxazoline) (NHS-POx) [144]. In the process, the NHS-POx was coated onto the gelatin patch. The hemostatic efficacy of the designed hemostatic patches was compared directly with commercial hemostatic patches, i.e., Hemopatch^®®^ and Tachosil^®®^ [144]. In vivo studies in liver and spleen injury models in a heparinized pig demonstrated that POx-NHS-coated gelatin patches showed a similar hemostatic response as compared to Hemopatch^®®^ [144]. However, the POx-NHS-coated patches proved superior on activation of the natural coagulation cascade. The hemostatic efficacy of the polymer was found to depend on the coating homogeneity and density. Furthermore, the presence of NHS-ester, as well as the hydrophilic group, is required to ensure the optical hemostatic response [144]. Bone hemorrhage is also a severe issue in the medical field. Bone wax is used to stop the bleeding from damaged bones and is generally smeared onto the bleeding edge of the bone, which immediately seals the hole and provides hemostasis through a tamponed effect. In 1984, Mattie et al., used a polydioxanone (PDS) polymer to control bone bleeding [145]. Biocompatibility, low molecular weight, and putty-like consistency at room temperature of PDS make it a suitable hemostatic bone sealant. In a novel attempt, Marin et al., have developed collagen-polyvinyl alcohol-indomethacin biohybrid matrices for wound dressings. Their study showed that collagen sponges become more compact with polyvinyl addition. Through their experiments, they found the most stable matrix to be the one with a 3:1 collagen to polyvinyl ratio. They also incorporated indomethacin, an anti-inflammatory drug, in the matrices for in vitro drug release [146].

Also gaining popularity is the synthesis of cryogels as hemostatic agents. Li et al., have first reported the potential of cryogels in tissue adhesion and wound dressing by developing a multifunctional antibacterial tissue-adhesive cryogel based on chitosan and polydopamine, exhibiting excellent mechanical strength and easy removability [71]. Similarly, Hou et al., have also reported their findings on polysaccharide-peptide cryogels made with glycol chitosan and polylysine for superior antibacterial properties [88]. Huang et al., have prepared dry cryogel hemostats with excellent clotting properties by crosslinking gelatin and polydopamine [147]. From their results from a rabbit liver model, they were able to find that these cryogels showed better clotting capacity and adhesion compared to other gelatin hemostatic sponges. In another unique attempt, Zhao et al., have developed an injectable antibacterial nanocomposite cryogel with shape recovery for non-compressible hemorrhage [148]. The cryogel was synthesized with carbon nanotubes (CNT) and glycidyl methacrylate-functionalized chitosan. They were able to exploit the heavy crosslinking to achieve robustness while also using the conductivity imparted by the CNT’s, facilitating bacterial death by irradiation.

With recent advancements in technology, research has progressed to capacitate unique electrically and magnetically activated hemostatic systems. Hemostats engineered to be sensitive to electric and magnetic fields can be guided to deliver therapy-targeted sites. Shi et al., have reported a novel Fe_3_O_4_-microporous starch-based hemostat that can be guided to obscure, narrow, and bent wound channels to stop bleeding, instantly [149]. The hemostatic material is able to assemble into microparticle chains and move to the target site under the influence of an externally applied magnetic field [149]. In another study, Ziv et al., have been successful in synthesizing magnetic γ-Fe_2_O_3_ nanoparticles that can conjugate with thrombin at the blood-clot site, stabilizing it against anti-thrombin inhibitors [150]. According to their study, the physical conjugation of thrombin with these nanoparticles substantially improves its storage stability and shortens the effective blood-clotting time [150]. Magnetically activated systems can also play an indirect role in hemostasis, as shown by Davies et al. [151]. They have developed a colloidal system of methacrylate ester monomers, peroxide, and copper salts with magnetic nanoparticles that act as inhibitors. In the presence of an external magnetic field, these nanoparticles can be removed from the system, thereby triggering the copper-mediated polymerization reaction. This opens up new avenues for magnetically triggered reaction initiations at wound sites, and may be applied to hemostatic systems as well [151]. Luo et al., have developed an electric field-modified electrospinning technique that enables the precise deposition of nanofibers at wound sites [152]. They have shown that their electrically activated deposition process can initiate rapid hemostasis in organs, such as the kidney or liver, by precise in situ delivery of cyanoacrylate fibers [152]. Electric fields have also been shown to play an important role in wound healing [153]. Endogenous electric field-directed migration of keratinocytes is known to be an essential step in wound epithelialization. These electric fields are generated intrinsically in the body, and the application of external electric fields to promote this process has found considerable success. However, the translation of this technology in hemostatic systems holds huge potential and is yet to be fully explored [153]. Table 3 lists popular commercial polymer-based hemostatic systems today, along with a few issues and side-effects associated with their use.

## 3. Conclusions and Future Outlook

Inducing rapid hemostasis at the site of trauma remains a challenge for polymer-based hemostasis agents when the hemorrhage is massive, especially with exceptionally high bleeding rates (>90 mL/min.). Moreover, poor mechanical properties, such as weak adhesion strength and low mechanical durability, flowability of the polymeric formulation, and elasticity of the materials, are some of the crucial factors that can limit the applications of polymer-based hemostatic materials in the field. Therefore, the market for polymer-based hemostatic material is still young, with foci on reliable biosafety, high hemostatic efficiency, suitable mechanical strength, and high resilience, that can together achieve rapid hemostasis in a short time. Most of the hemostatic products have been researched and developed for use in military settings, wherein leakage of materials (*viz*., reactants, ingredients, polymers, nanomaterials) from the hemostatic agents and incomplete delivery of the hemostatic formulation can result in potential risks to the human body that can cause infections, and provide sites for tumorous cells and thrombus [161]. While organic-based hemostatic agents have attracted increasing attention in the field of hemostasis due to their wide range of sources, nontoxicity, hydrophilicity, air permeability, minimum side effects, and other advantages compared to synthetic polymer-based agents, they also have certain downsides [162]. For example, the hemostatic efficacy of natural polymer-based hemostatic agents needs to be built on because they still have lower potential efficacy than that of inorganic hemostatic materials, as reported by the United States Army Institute of Surgical Research (USAISR) [152].

Manufacturers have experimented with various methods of administering hemostatic agents to bleeding wounds. Some products are packaged into a granular form which can be poured directly into the wound, while some others act in the form of an adhesive foam. Each method of administration has distinct advantages and disadvantages depending on the location and type of injury being treated. Others are incorporated into a dressing or mesh, which allows the caregiver to apply direct pressure to the site of injury [163] Such dressings can be engineered either as a rigid bandage, a small bag, or a gauze, which must be unrolled prior to application.

Advanced products for the effective management of medical and surgical wounds include hemostatic agents, fibrin and other sealants, high-strength medical adhesives, and products that prevent post-surgical adhesions [164]. These products play active roles in accelerating and optimizing wound healing. Additionally, traditional products and technologies, *viz.* sutures, tapes, and more recently, staples, clips, and other mechanical wound closures, play a role in wound management that is largely limited to physically juxtaposing wound edges in order to enable the body’s natural wound-healing processes to occur across a minimal gap. The use of sutures, tapes, and other physical wound closures dates back to thousands of years ago, and while a great many wound types can be adequately managed by these devices, plenty of opportunity and promise remains for new products, whether used alone or adjunctively with traditional products, to accelerate wound healing, minimize blood loss, minimize infection, and otherwise reduce the overall cost of healing wounds.

**Figure 5:**
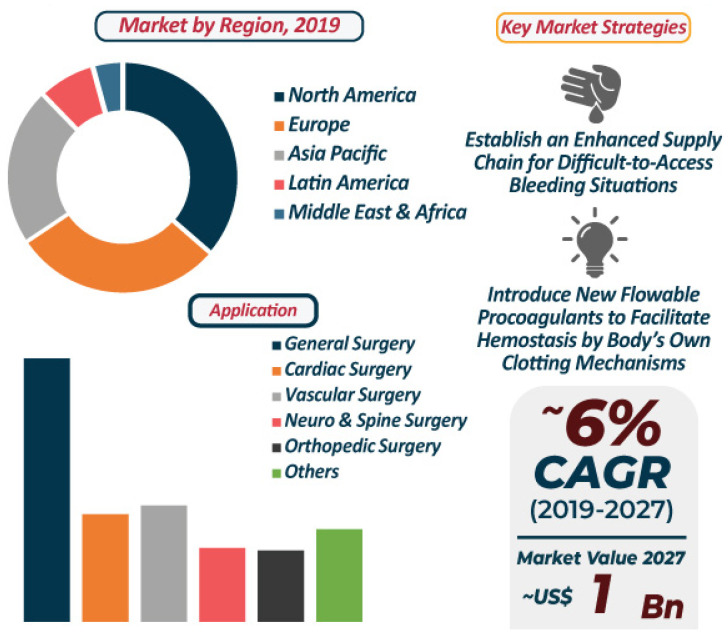
Flowable Hemostats market: 2019–2027 [165].

Hemostatic agents are always in high demand for prehospital hemorrhage control. Companies in the flowable hemostats market are constantly developing devices that work fast and improve visibility. The fast mechanism of advanced flowable hemostats helps in controlling bleeding from obscure sites in the shortest possible time. Gelatin matrix hemostats are gaining prominence in the market, as the total combined revenue of porcine and bovine gelatin materials is estimated to reach ~US$ 1 Billion by the end of 2027 [165]. Figure 5 represents a graphic illustration of the flowable hemostasis market projected from 2019 to 2027.

As such, the revenue of general surgery applications is the highest in the flowable hemostats market. Companies in the flowable hemostats market are striving towards introducing special applicator syringes in devices. The flowable hemostats market is expected to expand at a CAGR of ~6% during the assessment period. However, restriction of sales under federal US law remains a hurdle for med-tech companies to overcome. Stringent regulations can inhibit the growth of the US flowable hemostats market, which is anticipated to dictate the highest revenue among all regions in the market landscape. As a result, manufacturers have started increasing the availability of clinically proven flowable hemostats. Med-tech companies are increasing their R&D activities in flowable procoagulants that promote hemostasis by initiating the body’s clotting mechanism. They are expanding the availability of devices that eliminate the need for needles for mixing the materials. Companies are also aiming at popularizing hemostats that can be topically applied to control bleeding from vascular access sites and percutaneous catheters and tubes [165]. Other highly desirable parameters would include cost-effective solutions and formulations synthesized with FDA-approved materials that can conform to any wound dimension/geometry, are easy to use and replace, while remaining functional in inclement weather, dust, and at temperatures ranging from −40 to 50 °C, at least.

The needs and challenges associated with treating trauma wounds from arterial and venous injuries accompanied by severe bleeding require an immediate solution that can be used for relieving exsanguinations from external wounds resulting from accidents or operations. This necessitates the development of unique hemostatic treatments that can deliver the hemostatic agent in a few seconds and arrest bleeding within two minutes at the point of injury, without any intervention of manual pressure on the wounded area or requiring any kind of special training for their administration. The objective of a good hemostatic agent is to provide the body with a means to rapidly curb or stop bleeding from external and internal wounds related to single or multiple injuries involving arterial, venous, and/or parenchymal hemorrhage in a manner superior to those currently available. The major issues with the hemostatic agents include transmission of blood-borne pathogens, thromboembolic complications, and antibody response. There is also a requirement to develop more hemostatic agents that can remain functional for longer lengths of time without having to be replaced. On this basis, more studies are needed to analyze the performance closer to “real-world” situations, which often necessitates unique and personalized trauma and clinical settings. As such, extensive experimentation and clinical trials are required to be conducted, and significant research is still ongoing towards the quest of developing an infallible hemostatic bandage.

## Figures and Tables

**Table 1:** Hemostatic functions of natural polymeric systems.

Name [Ref.]	Polymeric System	Mechanism of Action	Case Study Model	Findings
Chitosan [50,51,81,95,96]	Chitosan/*ε*-polylysine hydrogel Chitosan-nano-bioglass composite CMCS-TA-BDBA hydrogel PVA/CSENDHM nanofibrous membrane.	Employed for hemostasis, in the form of powder, films, sponges, hydrogels, particles, fibers. Powders: Manual compression, as well as the interaction with erythrocytes, provide rapid coagulation. Hydrogels: Facilitates barrier formation and prevents blood flow from the cavity.	In vivo study of acute liver puncher models in rats, rabbits, and pigs.	Anti-microbial and anti-bacterial activities; excellent adhesion ability; rapid blood coagulation; high water absorption; biocompatible.
Collagen [97]	CollaStat^®®^ (Collagen and thrombin) and Floseal^®®^ (Gelatin and thrombin)	Provides a site for platelet adherence, activation, and aggregation. The activated platelets agglomerate around the wound and stop the blood flow.	The hemostatic efficacy of CollaStat^®®^ and FloSeal^®®^ have been compared in a rabbit jejunal artery injury model.	The mean hemostasis time for CollaStat^®®^ was found to be significantly shorter compared to Floseal^®®^ (64.0 ± 0.5 s vs. 84.0 ± 7.8 s).
Dextran	Aldehyde dextran sponge	Accelerates coagulation by rapid wound closure, cells’ initiation, and aggregation, as well as coagulation factors’ aggregation on the wound site.	In vivo study of the femoral artery and liver injuries model in rabbits.	Low cytotoxicity; remarkable blood loss; quick blood absorption and strong tissue adhesion.
Gelatin [56,57,58,98]	FloSeal^®®^ (Gelatin-thrombin granules). Graphene oxide-gelatin aerogels. Curcumin-alginate-gelatin sponge. m-TG-thrombin-gelatin	Gelatin granules swell when they come in contact with the blood. These swollen granules stop hemorrhage by blocking the bleeding site.	In vivo (liver and spleen rupture model in swine, rat liver trauma model, liver abrasion model in rabbit). In vitro (platelets adhesion, whole blood-clotting time, total blood absorption).	Excellent clot integration into the surrounding tissues; safe to implant in the body; reduced blood-clotting time; potential for preventing tumor recurrence.
Alginate [65,67,99,100]	PGA/alginate/AgNP Alginate fibers Alginate-PHMB-AgNP polyamide nanocomposites Hydroxy apatite/alginate granules.	Calcium ions are released in exchange for sodium ions when calcium alginate comes in contact with blood. These released calcium ions promote prothrombin activation in the clotting cascade which leads to rapid hemostasis. Alginate granules swell enough to block the bleeding site.	The alginate-based hemostatic dressing efficacy was characterized by blood-clotting time. Biocompatibility of the dressings was studied using degradation weight change.	pH-sensitive swelling properties; excellent hemostatic performance; anti-bacterial properties.
Cellulose [49,70,74,101]	Cellulose-modified chitosan foam sponge. Oxidized cellulose patch (Surgicel^®®^).	Facilitates hemostasis by quickly absorbing liquid, entrapping platelets and erythrocytes, and increasing blood coagulating factors.	Rabbit femoral artery injury model. Mouse tail amputation model.	Excellent water-absorbing ability, improved mechanical strength; low hemolysis rate, benign cytotoxicity; good resilience ability; superior hemostasis; good candidate for chronic wound treatment.
Hyaluronic acid [32,59,82,94,102]	GelMA-HA-NB hydrogel HA/gelatin hydrogel HAPPI HA-Serotonin hydrogel	HA-based hydrogels act as tissue sealants for hemorrhage control.	In vivo (rat femoral artery bleeding model, liver bleeding rat model, mouse tail bleeding model, mouse abdominal wall abrasion model).In vitro (shear test, adhesion test, compression test, total blood-clotting time test)	Shorter gelation time (< 2 min), good stability; strong burst strength; excellent sealant strength; improved hemostatic capability; potential application as a trauma wound sealant.
Starch [90,93,94,103,104]	*k*CA-coated starch/cellulose nanofibers St-Dopa hydrogel SPS-STMP hydrogel TRAP-Starch-PEG sponge	When Ca^2+^ CPSMs are applied to the bleeding sites, it provides sites for RBCs and platelets’ adhesion, and forms gel-like matrices which block the irregular bleeding. Starch-based sponge provides pressure to the wound and promotes hemostasis.	In vivo mouse tail amputation method; rat tail bleeding model; rat liver laceration model.	Hydrogels: rapid sol-gel transition; good swelling ratio; excellent cyto/hemocompatibility. Sponge: high resilience; good mechanical strength; high expandable; useful as a topical hemostatic agent for uncontrolled and non-compressible hemorrhage.

m-TG—microbial transglutaminase; GelMA—methacrylate gelatin; NB—butanamide; *κ*CA—kappa carrageenan; St—Starch; SPS—porous starch; STMP—Sodium trimethaphosphate; AgNP—Silver nanoparticles; PHMB—Poly(hexamethylene) biguanide; CMCS–*O*-carboxymethyl chitosan; TA—Tannic acid; BDBA—1,4-benzenediborinic acid; PVA—Polyvinyl alcohol; CSENDMH—quaternary ammonium *N*-halamine chitosan.

**Table 2:** Hemostatic functions of synthetic polymeric systems.

Synthetic Polymer	Synthetic Polymeric System [Ref.]	Mode of Application	Case Study Model	Time to Achieve Hemostasis	Findings
Polylactic co-glycolic acid (PLGA)	TissuePatchDural^TM^ [107] G-CSF-dextran nanoparticle-PLGA [108]	Adhesive patch Gauze	Patients underwent an intradural neurosurgical procedure. Femoral artery model in rats.	1 min -	Excellent for postoperative cerebrospinal fluid (CSF) leakage; no foreign body reaction. Provides hemostasis; increases neutrophil activity.
Polycaprolactone (PCL)	Gelatin/PCL [110] Chitosan/PCL/Gelatin [111] PCL/Starch [112]	Nanofibrous matrix sheet Composite scaffold Mat	In vivo rat liver injury model. In vitro whole blood clotting. Blood-clotting time was determined using the modified Lee and White method.	- - -	Safe and effective hemostat; helps in liver regeneration. Strong blood coagulation ability; prevent cell infiltration; biocompatible. Good hemostatic potential with a faster blood-clotting rate.
PolySTAT	PolySTAT [131] PolySTAT/Chitosan [135]	Gauze Injectable polymer	Rat femoral artery injury model. Trauma and fluid resuscitation model in rat.	- -	Rapid blood adsorption; withstand arterial pressure. Stabilizes fibrin clot, assists in the formation of a strong clot, mimics the function of transglutaminase factor XIII.
Siloxane	Siloxane-based mixtures [136]	Semi-solid gel	Porcine model	-	Semisolid matrix forms an artificial blockage to control bleeding.
Polyethylene oxide (PEO)	CMC-PEO-KC [137]	Granules	Femoral artery model in rats.	90 s	CMC-PEO-KC hydrogels are capable of clotting whole blood, adhering to platelets, and accelerating clotting time.
Polyacrylamide (PAM)	Keratin-PAM [138]	Sponge	Rat penetrating liver trauma model	8 mm wound—48 s 11 mm wound—57 s.	Highly expandable upon blood adsorption; useful in trauma application.
Polyethylene glycol (PEG)	HA-PEG [114] Chitosan-PEG [117] PEG-NHS [140]	Hydrogel Hydrogel Hydrogel	Laceration model in rabbit liver and pig skin. Liver penetration model in rat. Hemorrhaging liver mouse model; rat skin incision model.	30 s - 5 s	Rapid hemorrhage control; prevent from infection; good candidate for first-aid treatment of critical wound. Rapid hemostasis along with accelerated wound healing; excellent swelling and mechanical properties; low cytotoxicity. Excellent bioadhesive hydrogel; excellent hemostatic ability; possible alternative for sutures.
Polyurethane [106]	PU-chitosan [141]	Foam	Rat tail tip model	PU—23.9 min PU-chitosan—21.5 min	Possible alternative for topical hemostatic agents, chitosan added to PU decreases the bleeding time.
Cyanoacrylate	Octyl-cyanoacrylate [142]	Hydrogel	Porcine epistaxis model in pigs.	259 s	Cost-effective. No follow-up required.
Polyethylene terephthalate	Oxygen- and nitrogen-treated PET coated with heparin [143]	-	In vitro study characterized by platelet adhesion in whole human blood using optical imaging techniques.	-	Oxygen-functionalized PET shows better hemostasis compared to nitrogen-functionalized PET.
Poly-2-oxazoline (POx)	POx-NHS [144]	Powder	Liver and spleen injury model of profuse bleedings in heparinized pigs.	20–25 s	NHS-ester and hydrophilic groups required for better hemostatic application.
Polydioxanone (PDS)	PDS + Sesame oil/Castor oil/Almond oil/Carbowax 400 [145]	Putty	Rat penetrating liver model	-	Can be used as a bone sealant; effective to osseous hemorrhage; no irritation.

G-CSF—Granulocytes-colony-stimulating factor; CMC—Carboxymethyl cellulose; KC—Kappa-carrageenan; NHS—*N*-hydroxy succinimide ester.

**Table 3:** Commercially available polymer-based hemostatic systems.

Name [Ref.]	Polymeric System	Form of Application	Hemostatic Efficacy (Based on Clinical Trials)	Reported Drawbacks
GelFoam^®®^ [41]	Gelatin	Compressed sponge	Capable of absorbing up to 45 times its weight of whole blood. Hemostatic success in 10 min.	Abscess formation, breathing difficulties, fluid encapsulation.
Tachosil^®®^ [154]	Equine collagen	Two-layer patch/sponge material with equine collagen on one side and fibrinogen-thrombin on the other side.	Hemostasis achieved in 3 min.	Hypertension, increased transaminases.
Surgicel^®®^ [154,155]	Cellulose (ORC)	Loose knit absorbable powder	Hemostatic success in 5 min.	Foreign body reactions, tissue necrosis, nerve damage.
Traumastem^®®^ [156]	Cellulose (ONRC)	Fibrous re-absorbable dressing	Hemostatic success in 10 min.	No adverse reactions reported.
InStat^®®^, HeliStat^®®^ [154,157]	Bovine collagen	Dry absorbent hemostatic agent in microfibrillar form.	Hemostatic success in 5 min. Left in place, reabsorbed within 8 to 10 weeks.	Swelling and allergic reactions.
FloSeal^®®^ [154,158]	Gelatin	Adjunct hemostat–collagen granules dispersed in human thrombin (syringe application).	Hemostatic success within 10 min. Forms mechanically stable clot. Reabsorbed within 8 to 10 weeks.	Rare reports of inflammatory responses.
Celox Rapid^®®^ [11] Duraseal^®®^ [154] TissuGlu^®®^ [159] Tegaderm^®®^ [160]	Polyurethane Chitosan Polyurethane PEG	Adhesive mesh. Hemostatic gauze. Adhesive sterile dressing. Absorbable hydrogel delivered by a dual-syringe applicator.	Adhesive crosslinking takes place in 30 to 40 min, allowing surgeons to reapproximate skin layer before the adhesive sets in. Can eliminate the need of post-surgical drains. Stops bleeding within one minute of compression. Stops bleeding within one minute of compression. Crosslinks immediately and creates watertight closure within 5 min.	Seroma formation, hematoma, immunological reactions, wound separation. Embolism, pain. Embolism. Pain. Renal compromise, inflammatory reactions, delayed healing.

## Data Availability

Not applicable.
